# Deep learning based phenotyping of medical images improves power for gene discovery of complex disease

**DOI:** 10.1038/s41746-023-00903-x

**Published:** 2023-08-21

**Authors:** Brianna I. Flynn, Emily M. Javan, Eugenia Lin, Zoe Trutner, Karl Koenig, Kenoma O. Anighoro, Eucharist Kun, Alaukik Gupta, Tarjinder Singh, Prakash Jayakumar, Vagheesh M. Narasimhan

**Affiliations:** 1 Department of Integrative Biology, The University of Texas at Austin, Austin, TX USA; 2 Department of Surgery and Perioperative Care, Dell Medical School, Austin, TX USA; 3 Department of Biomedical Engineering, The University of Texas at Austin, Austin, TX USA; 4 The Department of Psychiatry at Columbia University Irving Medical Center, New York, NY USA; 5 The New York Genome Center, New York, NY USA; 6 The Mortimer B. Zuckerman Mind Brain Behavior Institute, New York, NY USA; 7 Department of Statistics and Data Science, The University of Texas at Austin, Austin, TX USA

**Keywords:** Genome-wide association studies, Computer science

## Abstract

Electronic health records are often incomplete, reducing the power of genetic association studies. For some diseases, such as knee osteoarthritis where the routine course of diagnosis involves an X-ray, image-based phenotyping offers an alternate and unbiased way to ascertain disease cases. We investigated this by training a deep-learning model to ascertain knee osteoarthritis cases from knee DXA scans that achieved clinician-level performance. Using our model, we identified 1931 (178%) more cases than currently diagnosed in the health record. Individuals diagnosed as cases by our model had higher rates of self-reported knee pain, for longer durations and with increased severity compared to control individuals. We trained another deep-learning model to measure the knee joint space width, a quantitative phenotype linked to knee osteoarthritis severity. In performing genetic association analysis, we found that use of a quantitative measure improved the number of genome-wide significant loci we discovered by an order of magnitude compared with our binary model of cases and controls despite the two phenotypes being highly genetically correlated. In addition we discovered associations between our quantitative measure of knee osteoarthritis and increased risk of adult fractures- a leading cause of injury-related death in older individuals-, illustrating the capability of image-based phenotyping to reveal epidemiological associations not captured in the electronic health record. For diseases with radiographic diagnosis, our results demonstrate the potential for using deep learning to phenotype at biobank scale, improving power for both genetic and epidemiological association analysis.

## Introduction

For most complex disease traits, clinical endpoints are usually binary (case–control) in nature. In particular, data on disease outcomes from population-scale biobanks are only available through recorded ICD-10 billing codes or self-reported diagnosis^1–3^. While these datasets have provided invaluable insights into the genetic basis of disease, case ascertainment based solely on information available in the electronic health record (EHR) or from self-reports can be biased by a multitude of factors, including differences in how patients were billed^4^, differential diagnosis due to assessment by clinicians (non-specialist vs specialist)^5^, or differences in classification or diagnosis based on disease severity^6^.

An alternate approach to ascertaining disease status might be to directly perform clinical-grade assessment from a patient’s medical images using a consistent diagnosis protocol. However, this is difficult to achieve at large scale such as in population biobanks where sample sizes range from hundreds of thousands to millions of individuals^1^. For musculoskeletal diseases such as knee osteoarthritis (OA), radiography is the routine course of diagnosis in the clinic and the primary means to assess disease progression through sclerosis, osteophytosis (bone spurs), and narrowing of the space between the femur and tibia (knee joint space)^7^. For such radiographically diagnosed diseases, computer vision approaches for automated phenotyping based on training data from clinicians offer the potential to ascertain both case status and disease severity at scale. Such approaches have already been used for determining pneumonia and SARS-CoV-2 cases from chest X-ray images, with reported accuracy even higher than expert radiologists based on ground truth from molecular information^8,9^.

Taking advantage of these developments in computer vision, recent genetic studies have successfully applied deep-learning methods to generate image-derived phenotypes (IDPs) of body fat distribution, heart structure, liver fat percentage, and brain morphology, and have linked them with genome-wide significant loci^10–14^. While some recent studies on musculoskeletal disease employ these novel phenotyping approaches^15–17^, neither these nor the studies on other traits have specifically investigated how generating quantitative IDPs that underlie binary disease status could be used to improve power for gene discovery at biobank scale.

Quantitative measurements which provide information about variation in the severity of the progression of the disease are already routinely utilized in predicting an individual’s risk for complex disease in the clinic. For example, LDL cholesterol levels are a quantitative biomarker measured in blood samples, as a primary biomarker to assess risk for myocardial infarction, one of the leading causes of death worldwide^18^. Multiple lines of functional evidence suggest that LDL cholesterol levels are also causally linked to heart disease and lowering LDL levels over an entire lifetime through the use of statins is the most widely used long-term prescription medication^19^. In theoretical work, it has been demonstrated that with equal sample size and when the proportion of cases in a case–control design is equivalent to the prevalence of the disease in the population, the power of a case–control association study is considerably lower than that of a quantitative association study. This is in part because key information about variation in the trait in the sample population is lost when transforming a continuous trait into a binary one^20^.

Building on these foundational ideas, in this work we first trained a binary classification model to identify knee OA cases at clinician-level performance and deployed this at biobank scale to compare our radiographically obtained results to the ICD-10 record. Second, we trained an image segmentation algorithm to obtain a quantitative measurement highly correlated with knee OA severity, minimum joint space width (mJSW), to examine differences in power between genome-wide association studies (GWAS) carried out using quantitative approaches versus a case–control design. Third, we generate a polygenic risk score (PRS) for each phenotype to evaluate if improvements in statistical power to find novel loci translate to better prediction of ICD-10 record knee OA (M17) in a hold-out dataset of over 300,000 individuals. Finally, we examined epidemiological associations to link our IDPs with fractures, an outcome of major clinical relevance.

## Results

### Dataset and quality control of DXA imaging and genetic data

To study the genetic basis of knee phenotypes, we jointly analyzed paired dual energy X-ray absorptiometry (DXA) imaging and imputed genome sequence data of 42,284 individuals in the UK Biobank (UKB). We first restricted the dataset to individuals of white, British ancestry, applied standard variant and sample quality control (QC), and analyzed 12.1 million common biallelic SNPs with minor allele frequency greater than 0.1%^1^ (“Genetic QC”). Next, as the bulk imaging data from the UKB consisted of DXA images that reflect scans of different body parts, we used a deep-learning approach^15^ to subset the imaging dataset to only anterior-posterior (AP) view knee scans. We then removed individuals that had outlier image resolutions or poor quality DXA scans, and padded images to a standard size for processing (see “Image segmentation, phenotype measurement and quality control”). Post quality control, we were left with combined imaging and genetic data for a total of 29,257 individuals aged between 46 and 81 with a mean age of 64. The ratio of males to females was 50.2% (*n* = 14,676) to 49.8% (*n* = 14,581) of the participants used in this analysis, consistent with the overall distribution in the UKB (see “UKB participants and dataset” and Table 1).

**Table 1:** Relevant population characteristics for the subset of participants used in GWAS, heritability, and PRS analyses.

Population characteristic	Mean (s.d.)
Participants, total	29,257
Age at DXA, years	64 (7.5)
Height, cm	169.5 (9.2)
BMI	26.5 (4.3)
On steroid medications, total (%)	424 (1)
Past knee trauma, total (%)	351 (1)
Male, total (%)	14,676 (50.2)
Age at DXA, years	64.6 (7.6)
Height, cm	176.2 (6.6)
BMI	27.0 (3.9)
Female, total (%)	14,581 (49.8)
Age at DXA, years	63.0 (7.3)
Height, cm	162.8 (6.2)
BMI	26.0 (4.6)
Bone mineral density, total	27,312
Legs, g/cm^2^	1.2 (0.18)
Body, g/cm^2^	1.2 (0.15)

Values are presented as the mean and standard deviation unless otherwise specified as the total participants and percent in the population. A subset of individuals (*n* = 1945) do not have bone mineral density calculations for either legs or the body.

### Automated phenotyping of knee OA achieves clinician-level performance

**Fig. 1:**
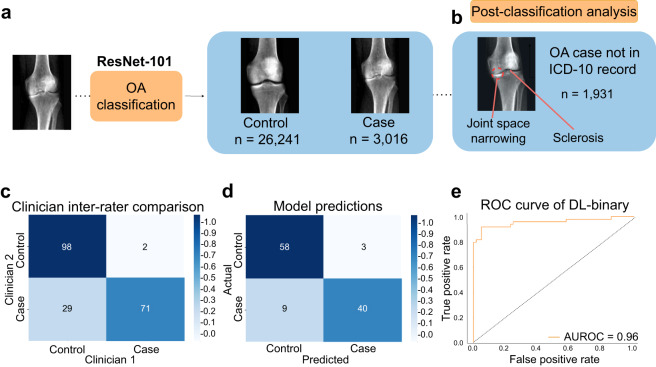
A deep-learning process for automated phenotyping of radiographic knee OA. **a** ResNet-101 based classifier for binary classification of knee OA, showing an example of a typical individual diagnosed as a case compared to a control individual. **b** Post-classification analysis using highlighted regions of the knee that are discriminatory for knee OA. We confirmed joint space narrowing and sclerosis of the bone (important features for case classification) are present in cases not reported in the ICD-10 record but identified by the model. **c** Inter-rater comparison of three clinicians grading AP view knee DXA scans expressed as a proportion out of 100. Color scale reflects the proportion out of the total for cases and controls. **d** Confusion matrix showing performance of the DL-binary model on validation data, with color scale reflecting the proportion of correct case and control predictions out of the total (*n* = 110). **e** Receiver-operating characteristic (ROC) curve for DL-binary, showing the performance of the model under different classification thresholds. AUROC area under the ROC curve.

To perform automated phenotyping for knee OA based on radiography, we used a binary classification approach based on the Kellgren-Lawrence (KL) grading system^21^ (usually graded 0–4, where a 4 is considered the most severe case of radiographic OA) to determine case or control status for each individual reflecting different levels of joint space narrowing, subchondral sclerosis, and the presence of osteophytes. Cases were considered individuals with a KL grade of 3 or higher—severe enough that annotating clinicians would consider a candidate for joint replacement surgery in the clinic. Controls were considered individuals who would not be candidates for joint replacement—a grade 2 or lower (see “Binary classification: DXA scan annotation procedure”). To train the deep-learning model, we obtained case–control assessment on 546 images based on the annotations of three board-certified orthopedic surgeons who independently assessed each image. We then split the dataset so that 80% (436 images) of the data was used for training and 20% (110 images) was used for validation. First, we compared several architectures and found the performance of the ResNet-101 architecture the best for our task (see “Binary classification: model selection” and Fig. 5). We next trained a binary classifier (which we refer to as DL-binary) using transfer learning with the ResNet-101 architecture^22^ (see “Binary classification: Network architecture and model training” and Figs. 1d, e and 6). The sensitivity and specificity of our model on the validation data (that is not used as part of the training process) was within the range of the sensitivity and specificity obtained between three clinicians grading the same set of images (clinician sensitivity: 0.77 ± 0.05, DL-binary sensitivity: 0.82 ± 0.07 Clinician specificity: 0.97 ± 0.05, DL-binary specificity: 0.95 ± 0.06) (Fig. 1c, d). The DL-binary model achieved an AUROC and AUPRC of 0.96, reflecting the model’s effectiveness in identifying true positives while minimizing false positives (Fig. 1e). We further validated the interpretability of the DL-binary model’s case–control assessment through an additional Grad-CAM analysis (see “Binary classification: Grad-CAM analysis for interpretation of model predictions” and Fig. 7).

### Image-based phenotyping reveals twofold more cases compared with ICD-10 records

We next deployed our trained model on the remaining 28,725 images of knee DXA scans from the dataset. We considered an individual a “case” if our model predicted the individual to have knee OA on either the left or the right knee and a control otherwise, in line with the ICD-10 code M17 for knee OA. We then assessed how many cases were determined by our deep-learning-based binary classification of radiographic OA as compared to what already exists in the ICD-10 code M17. We found that after deploying the DL-binary classifier, we determined 1931 more cases compared with the ICD-10 code for knee OA (ICD-10 code M17 1085 cases, DL-binary 3016 cases) (Fig. 1a). To provide additional support for cases reported by DL-binary that were not already reported in the ICD-10 code M17, our clinical team examined 100 individuals manually and confirmed the presence of osteophytes, reduced joint space and in some cases subchondral sclerosis (Fig. 1b). As these alone may not be diagnostic, we also investigated associations with three self-reported measures of knee pain in the UKB: knee pain experienced in the past month (binary), knee pain for 3+ months (binary, and reflecting knee pain experienced over a long duration) and rating of knee pain in the past three months (scale from 0 to 10). We found that in individuals who were newly identified as cases, the rate of self-reported knee pain was significantly higher compared to control individuals (individuals not diagnosed by ICD-10 code M17 or by DL-binary) across all three measures we examined (recent pain reported as knee pain in the past month: 49.4% in cases and 27.2% in controls, chi-square statistic = 536.6, *P* = <2.2 × 10^−16^, chronic pain as determined by knee pain lasting 3 or more months: 80.4% in cases and 70.6% in controls, chi-square statistic = 28.29, *P* = 1.5 × 10^−7^ and severity of pain reported in the last 3 months: mean rating of 3.33 in cases and 2.58 in controls, *t* test *P* = 1.45 × 10^−15^). These results suggest that knee OA is likely underdiagnosed in the ICD-10 record and that our approach is capable of identifying additional true cases not already present in the EHR.

### Image segmentation to measure joint space width

**Fig. 2:**
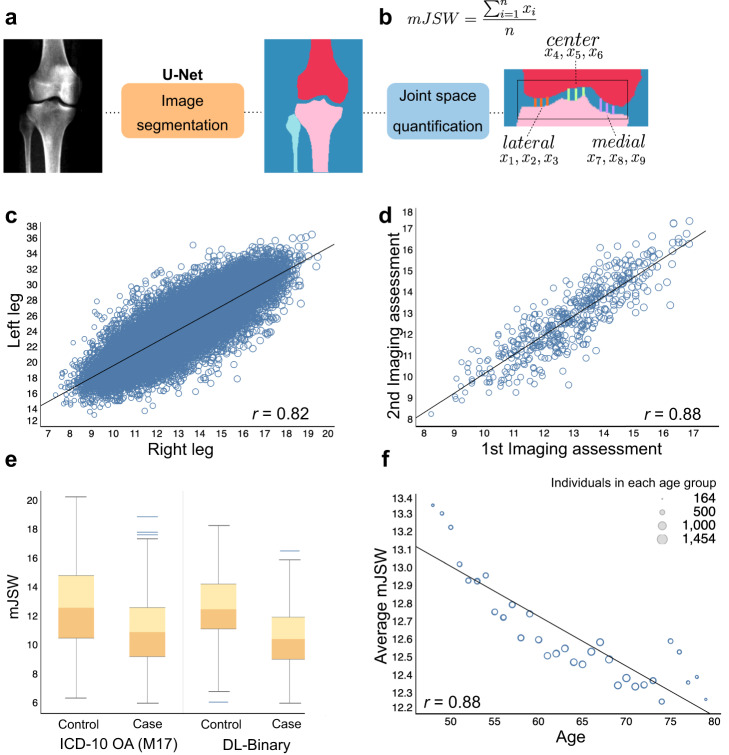
Deep-learning-based image segmentation for minimum joint space width (mJSW model). **a** Deep-learning-based image segmentation labeling the femur, tibia, fibula, and background based on a U-Net architecture (mJSW model). **b** Measurement computed from the mJSW model, taken between the tibia and the femur as the average of three points each in the lateral, center and medial portions of the knee joint. **c** Correlation of the mJSW phenotype between the right and left leg of the same individual (*n* = 29,257). **d** Correlation in calculated mJSW phenotypes between the first and second imaging visit for the same individual (*n* = 461). **e** mJSW is narrower in cases compared to controls, using both ICD-10 code M17 and DL-binary case identification. **f** Average mJSW decreases significantly with increasing age (ages 48–79, *r* = 0.88, *P* value < 0.0001). Circle size corresponds to the number of individuals within each age group, with larger diameters equating to a higher sample size relative to smaller circles.

To examine knee OA severity beyond simple case–control assessment, we developed a method to obtain a quantitative measurement from knee DXA scans known to be highly associated with the disease: the minimum inter-bone joint space between the femur and tibia, which we refer to as the mJSW phenotype. To perform automated measurement on the UKB dataset, we first collected training data for 63 DXA scan-derived images of the knee (40 training, 23 validation). On each of these images, we labeled the positions of the femur, tibia, and fibula at pixel level, which were then validated by a team of clinicians. We then trained a deep-learning model based on the U-Net architecture^23^ with a 34-layer ResNet encoder^22^ to perform semantic segmentation of the femur, tibia, and fibula in each DXA image at pixel-level resolution (Fig. 2a). After quality control and image normalization (see “DXA scan image quality control and standardization”), we computed the mJSW phenotype by measuring the distance between the femur and tibia along multiple positions on the medial, lateral and center axes of the joint. We then computed the average of these distances for each leg (Fig. 2b). The mJSW measurement is defined as the smallest of the two averages for either leg, returning one phenotype measurement per individual. If the individual only had a right or left leg DXA scan, this was used as the mJSW phenotype measurement for that individual. To standardize mJSW measurements across image resolutions, we regressed each of the joint space lengths on the overall height of the individual (see “Image segmentation: measurement and quality control”).

We evaluated the performance of the segmentation model in several ways. First, the set accuracy, the correspondence between labeled data and annotation of the trained model on validation data, was 0.99. Second, the correlation between measurements taken between the right and left leg was 0.82 (Fig. 2c). Third, the correlation between images taken of the same person across two imaging visits was 0.88, despite changes in image resolution, scanner, technician, and imaging position, demonstrating that our mJSW phenotype measurement process is fairly consistent across biological replicates (Fig. 2d). We do not expect to see 100% concordance across these replicates as joint space width often can change in a period of more than 2 years particularly in older individuals, in part due to possible knee joint cartilage degeneration. Fourth, we examined the relationship between the mJSW phenotype and OA status, both using the DL-binary model and the ICD-10 code M17 case–control data (Fig. 2e). As expected, the mJSW phenotype was significantly lower in cases compared to controls regardless of which case annotation we used (*t* test *P* < 2.2 × 10^−16^, and *P* < 2.2 × 10^−16^ for DL-binary and ICD-10 code M17, respectively). Finally, we examined the relationships between the mJSW phenotype and age—which is known to be highly associated with knee degeneration (Fig. 2f). Again, as expected, we found that the mJSW phenotype decreased significantly with age (linear regression, beta = −0.028, *P* < 2.2 × 10^−16^).

### Genetic associations using image-derived phenotypes

**Fig. 3:**
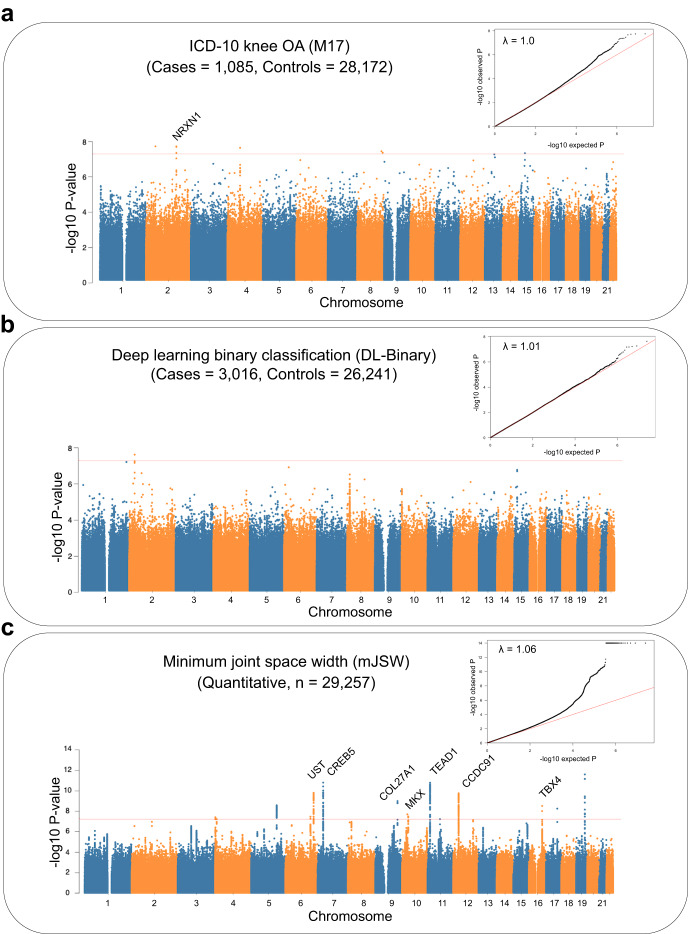
Manhattan plots for GWAS performed using three knee OA phenotyping methods. **a** ICD-10 code M17 defined knee OA case and control status. **b** The deep-learning-based automated case–control phenotype, DL-binary. **c** The deep-learning-based quantitative endophenotype, mJSW. Loci over the genome-wide significance threshold (t-statistic *P* = 5 × 10^−8^) that are in close proximity to only a single gene are annotated. All participants used in each of the three GWAS come from the same participant dataset (*n* = 29,257). Inset: Quantile–quantile (qq) plot of deviation of the observed *P* value from the theoretical distribution, along with the *λ* value quantifying genomic inflation.

Having obtained IDPs related to knee OA, we performed GWAS to link these phenotypes to their genetic basis. After generating summary statistics for each genetic association (Fig. 3), we estimated SNP heritability using LD Score regression^24^ for the three phenotypes: (1) knee OA as determined by the ICD-10 code M17 data from UKB, (2) knee OA as determined using DL-binary, and (3) mJSW, the quantitative phenotype highly correlated with severity of knee OA. The heritability of both binary phenotypes was low (ICD-10 code M17: 0.02 ± 0.02 and DL-binary: 0.04 ± 0.02). In contrast, the heritability of the quantitative phenotype mJSW was 0.24 ± 0.02. Genomic inflation for the three phenotypes also confirmed this trend, with lambda for ICD-10 code M17: 1.0, DL-binary: 1.01, and mJSW: 1.06. Deviations from expectation across the genome are visualized in the qqplots inserts on Fig. 3. We found 18 independent loci that reached genome-wide significance in the mJSW model GWAS, including one that was also significant in a previously reported GWAS for knee OA with 62,497 cases and 333,557 controls^25^ (Fig. 3). We annotated each of these 18 loci using the GWAS catalog, Human-mouse disease connection (HMDC) and Online Mendelian Inheritance of Man (OMIM) databases (see “Annotation of genome-wide significant loci”). We found one locus and six genome-wide significant loci with either binary phenotype respectively (DL-binary and ICD-10 code M17), though the mJSW and DL-binary model phenotypes had a genetic correlation of −0.92 ± 0.25 (see “Heritability and genetic correlation”). This suggests substantial improvements in power from using a continuous, quantitative measure associated with knee OA disease severity.

### Polygenic risk scores for knee joint space are highly predictive of knee OA

**Fig. 4:**
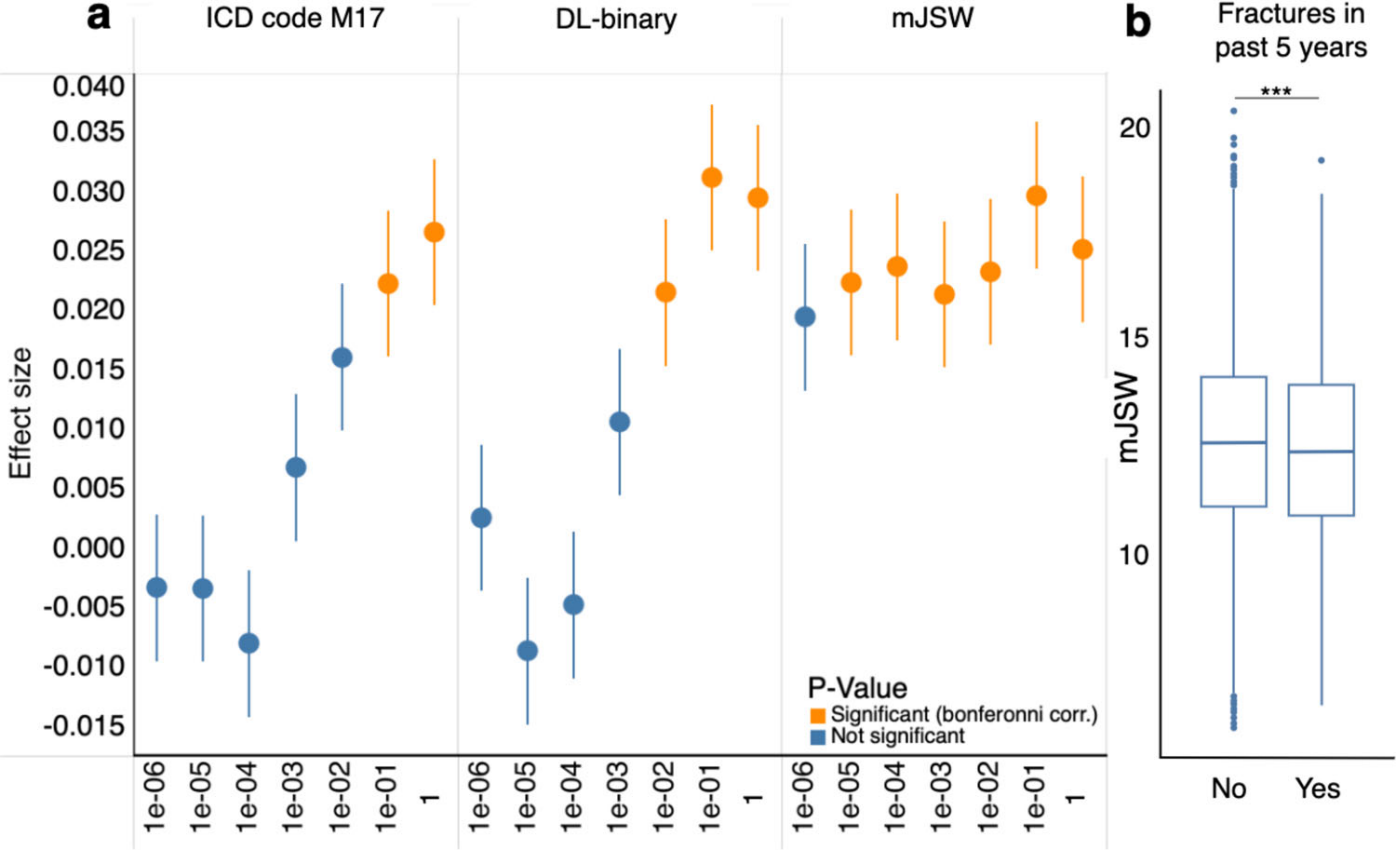
Genetic and epidemiological analysis of image-derived phenotypes. **a** Results of performing logistic regression analysis using the PRS generated from each GWAS to predict ICD-10 M17 diagnosis on a hold-out dataset of 371,686 UKB individuals, showing the regression estimate obtained at each *P* value threshold (error bars represent one standard error of the mean), colored by whether the test was significant after Bonferroni correction. **b** Boxplots showing the distribution of the mJSW endophenotype measurements in patients who experienced a fracture in the past 5 years (*n* = 24,147). Center line, bounds of box, whiskers and points correspond to the median, first and third quartile, lower and upper fence, and outliers. The mJSW phenotype PRS was significantly predictive of fractures in logistic regression analysis, asterisks correspond to *P* value significance (logistic regression, *P* < 1.10 × 10^−3^).

As our GWAS for the mJSW model identified many more loci of genome-wide significance compared to DL-binary or ICD-10 code M17, we wanted to assess if this translated to improved power to predict knee OA in individuals outside of our DXA imaged sample. We computed PRS using clumping and thresholding (selecting variants below different p-value thresholds ranging from 1 to 1 × 10^−6^) from the GWAS of the ICD-10 code M17, DL-binary and mJSW phenotypes, and deployed these scores on 371,686 individuals in the population who were not included in the GWAS (“Polygenic risk scoring and logistic regression”). We carried out logistic regression with binary presence or absence of ICD-10 code M17 diagnosed knee OA as the outcome, using z-scores generated from each of the PRSs as the predictor variable, and the first 20 PCs, age, sex, height, steroid medication use and past knee trauma as covariates (see “Polygenic risk scoring and logistic regression” and Table 3). After controlling for these variables and performing multiple hypothesis testing correction at the level of the total number of associations performed, the mJSW phenotype PRS remained independently associated with knee OA diagnosis at six of the seven total *P* value thresholds, while the DL-binary PRS or the ICD-10 code M17 PRS were only significantly associated with knee OA at fewer thresholds, again reflecting differences in power between the various GWASs (Fig. 4a).

### Quantitative phenotyping allows for novel epidemiological associations

In addition to improving power in genetic analysis, we wanted to examine if we could use the mJSW phenotype to improve statistical power to detect an important epidemiological outcome in the health record, fractures within the last 5 years. After controlling for height, sex, age, and body fat percentage, our results show that mJSW was significantly associated with fracture in the last 5 years (*P* = 1.10 × 10^−3^) in logistic regression analysis, but not with DL-binary (fractures: *P* = 0.79) or with ICD-10 code M17 (*P* = 0.171) (Fig. 4b). While previous work on a much smaller sample size of ~2000 individuals has shown that knee OA is associated with falls^26^, our results specifically implicate joint space narrowing with an independent increased risk of fractures, a known cause of death in individuals 65 and over^27^. These results emerge only upon examining our quantitative phenotype mJSW which captures an element of disease severity, revealing knee OA as an important risk factor for potentially fatal complications from fractures in older adults.

## Discussion

Here we report a deep-learning method to directly phenotype OA cases and controls (the DL-binary model), as well as joint space narrowing (mJSW model), from DXA scan-derived AP view knee radiographs of the UKB. We compare this image-derived phenotyping approach with case–control status of knee OA already available in the ICD-10 record code M17 on the same set of individuals, to determine if image-derived phenotyping approaches have an effect on statistical power in GWAS.

We find that the case–control phenotyping using the DL-binary classification method enables us to raise the case count almost twofold and circumvents some issues with sourcing cases from the EHR such as variation in specific definitions of OA or differences in a clinician’s perception of the disease^28^. While previous work has shown that the ICD-10 record can have issues identifying individuals with disease for a variety of reasons, our study carrying out image-based diagnosis at a large scale provides evidence of the extent to which the record can be incomplete.

In addition, both case–control methods lack information about disease severity, which may explain why they are underpowered compared to the quantitative measurement mJSW in the genetic and epidemiological analyses we investigate. The high genetic correlation between the mJSW and DL-binary phenotypes (92%) suggests that while the binary case–control phenotype of knee OA is underpowered compared with the quantitative mJSW phenotype, the genetic relationships found between the two phenotypes are consistent with one another.

While computer vision approaches to extract and analyze DXA scan-derived phenotypes are not themselves novel^16,17,29,30^, this work is amongst the first to use this approach on a disease for which diagnosis is primarily radiographic, to demonstrate that having a quantitative endophenotype that captures additional information about variation in disease severity improves power for genomic and epidemiological analysis. Although not based on the imaging data, two novel phenotyping methods leveraging deep learning to impute missing data in the UKB and to generate disease liability scores from binary case–control data in the EHR have shown significant boosts in statistical power for genomic studies^31,32^. Broadly, these and other approaches suggest that analysis of biobank data could benefit from quantitative refinement of disease phenotypes using alternative approaches.

One potential limitation of our study is that knee joint space narrowing is both causal and symptomatic in knee OA progression. As arthritis progresses, the joint space narrows due to the breakdown of cartilage, causing a resulting increase in pain and difficulty with movement. This narrowing of the joint space can also cause further damage, due to increased contact pressure at the affected joint. This makes it difficult to understand the root cause of knee OA with respect to the mJSW endophenotype, because joint space narrowing can both be a result of OA and a contributing cause to the progression of the condition. While the DL-binary method discovered approximately twofold more cases than what is annotated in the ICD-10 record, it is still likely to be an undercount due to our choice to use a particular instantiation of the model to limit the false positive rate as much as possible (Fig. 1e). Thus, despite improving the case–control ratio in the dataset, there may still be additional cases undetected by either method which could further improve statistical power in GWAS. Third, all GWAS in this work were restricted to individuals with European ancestry. Thus, the transferability of the specific findings in this genetic analysis (i.e., loci discovered from mJSW GWAS, trait heritability, and genetic correlation) across ancestries is not warranted without follow-up analyses.

Taken together, our study provides a proof-of-concept for the utility of quantitative phenotyping in biobank scale settings where a direct measurement of disease severity for a complex disease phenotype is possible. The results of this work suggest that this concept extends not only to other musculoskeletal diseases in which radiography is one of the primary methods for diagnosis (for example, directly measuring spinal curvature as opposed to scoliosis diagnosis), but to other analyses in which one can derive a quantitative alternative to case–control disease phenotyping.

## Methods

### UKB participants and dataset

All analyses were conducted with data from the UKB unless otherwise stated. The UKB is a richly phenotyped, prospective, population-based cohort that recruited 500,000 individuals aged 40–69 (mean 58) in the UK via mailers from 2006 to 2010^1^. In total, we analyzed data from 402,000 participants with genetic data of self-identified white British ancestry who had not withdrawn consent as of February 22, 2022. Of this genotyped cohort, 42,284 had available DXA images of either one or both knees. We removed individuals that had outlier image resolutions, poor quality DXA scans, or genotype missingness greater than 2.5%, resulting in a quality-controlled subset of 29,257 individuals. Relevant population characteristics are provided in Table 1. Access was provided under application number 65439.

### Dual-energy X-ray absorptiometry (DXA) imaging

The UKB has released DXA imaging data for a total of 50,000 participants as part of a bulk data field ID. The DXA images were collected using a Lunar iDXA instrument^1^ (GE Healthcare) in DICOM format. A series of eight images were taken for each patient: two whole body images—one of the skeleton and one of the adipose tissue, the lumbar spine, the lateral spine from L4 to T4, each knee, and each hip. Dual-energy X-ray absorptiometry (DXA) images were downloaded from the UKB bulk data. The bulk download resulted in 42,284 zip files, each corresponding to a specific identifier otherwise known as each subject’s EID. The uncompressed directories corresponding to each imaged subject contained several DXA images of the individual as described above. For this analysis, only images of the right and left knees from the AP view were used. It is important to note that all subjects in this analysis were instructed to lay flat on the DXA scanner machine during imaging, so that all resulting images are non-weight bearing.

### Phenotype and clinical data acquisition

The binary classification of patient disease phenotypes was obtained from a combination of primary and secondary ICD-10 codes. ICD-10 codes (FID 41270) were truncated to the initial three characters to increase sample sizes. Patients received a “one” if a disease code appeared in their hospital records, and a “zero” otherwise. Reports of a fracture within the last 5 years of any visit (instance 0 to 3) was considered a case. Our classification of fractures increases case counts while excluding any childhood incidence. The completeness of the knee OA ICD-10 code (M17) in the UKB may be subject to factors such as the quality of hospital episode statistics (HES) data, the accuracy of disease coding, and the representativeness of the UK Biobank cohort for knee OA^33^. These factors and others limiting the completeness of the ICD-10 record may impact the accuracy of the recorded diagnosis for knee OA used to validate the PRS generated from the DL-binary and mJSW GWAS. In total, for M17 there were 1085 cases and 28,172 controls. Of the controls, 4843 (17.2%) individuals have no ICD-10 diagnosis ever reported.

### Computing infrastructure

We carried out all training using the Python programming language (version 3.7.7) with the PyTorch^34^ and Fastai version 1^35^ libraries on NVIDIA 1080-TI GPUs on the Maverick2 system and NVIDIA Quadro RTX 5000 GPUs on the Frontera system of the Texas Advanced Computing Center using the CUDA 11.1 toolkit.

### DXA scan image quality control and standardization

DXA images in DICOM format were first organized by anatomy following the manifest files located in each directory output by the imaging machine. DXA scans were subject to further quality control following the methods described in ref. ^15^. Following initial data cleaning, AP view knee DXA scans were converted from DICOM to JPG format using the pydicom library^36^. To prepare a uniform set of images for segmentation, the numpy^37^ and opencv-python^38^ libraries were used to pad images to a standard width and height (800 × 1000 pixels), and outlier images that had resolutions outside of this standardized range were removed from all downstream analyses. Padded images were subject to further image resizing during training of the U-net architecture^23^ for segmentation (using a progressive resizing technique), but not during training of the classification model.

### Binary classification: DXA scan annotation procedure

The KL grade^21^-based phenotype (DL-binary) was defined taking the following observations as input: presence or absence of osteophytosis, visible sclerosis of bone, and narrowing of the inter-bone joint space between the femur and tibia). Participating surgeons were instructed to annotate images as 0 or 1 based on whether or not each image qualified as KL grade 3 or greater, meaning that based on the radiographic evidence of knee OA the individual would be a candidate for joint replacement surgery. We considered 0 to be a control (but not necessarily devoid of any radiographic OA symptoms) and 1 to be a case (KL grade 3 or 4) warranting joint replacement.

We obtained case–control assessments of 546 AP view knee DXA scan images from three board-certified orthopedic surgeons. DXA scan images were selected from the UKB with reference to the ICD-10 code M17 to create an approximately balanced dataset for training and validation. We used a consensus grading approach; first, three pairs of physicians graded a third of the total dataset (182 images for each pair). Second, all three physicians then met together to review the labels produced from the pairwise grading. The binary case–control diagnoses generated from the consensus grades output from this process were used as ground truth diagnoses for training the DL-binary model. 436 images (80% of the data) were used for training and 110 images (20% of the data) were randomly sampled and reserved for validation (not seen by the model during training).

To understand the inter-rater reliability among the three surgeons, we computed the average across all three physicians used to label the training data. Three sets of 182 images from this dataset were used for clinician inter-rater comparative analysis. We expressed the contingency tables produced from each pair as a proportion out of 100, and the resulting confusion matrix representing the inter-rater reliability among the three physicians is an average across these three tables (Fig. 1c).

### Binary classification: normalization and data augmentation

Prior to performing binary classification, images were scaled to 224 × 224 pixels and normalized using ImageNet statistics. The ResNet-101 convolutional neural network (CNN) weights were initialized using the Kaiming normal method^22^. While training, multiple transformations were applied to the input images to regularize the model. These included a padding process as described above, as well as other transformations such as vertical flipping of the image, random rotation, zooming, warping, light, and contrast change. This data augmentation was performed to improve the model’s ability to generalize in its predictions relative to variation in contrast and other image artifacts common to DXA scanning^39^.

### Binary classification: model selection

**Fig. 5:**
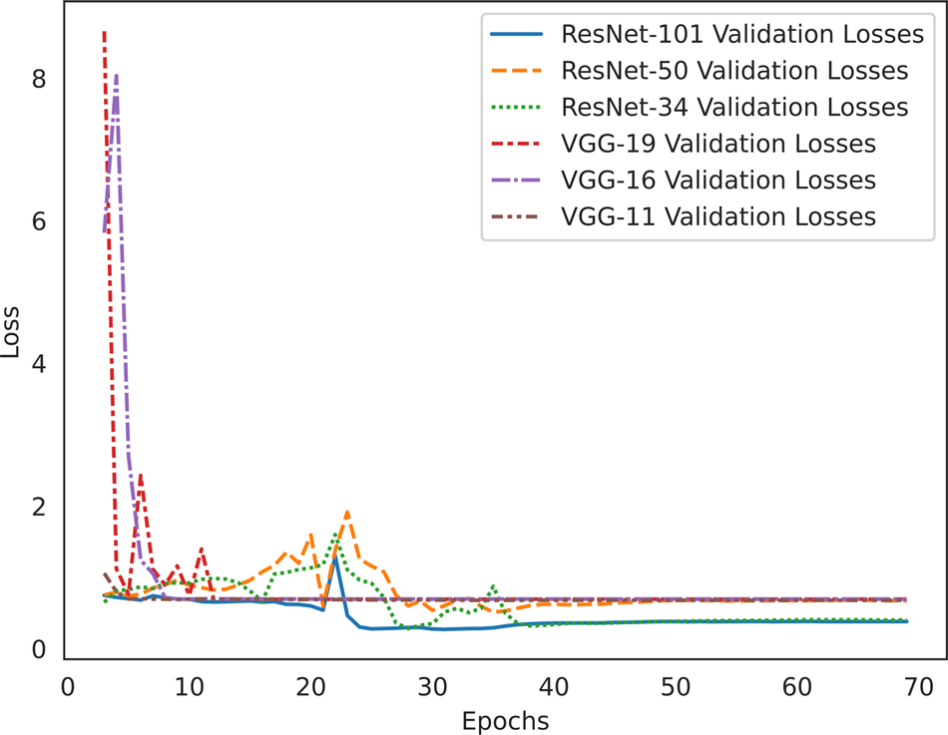
Validation loss per epoch for benchmarking six deep-learning architectures. Validation loss (*y* axis) versus number of epochs (*x* axis) for six deep-learning algorithms (VGG-11, VGG-16, VGG-19, ResNet-34, ResNet-50, and ResNet-101) during model benchmarking. The plot demonstrates the performance of each algorithm in terms of loss minimization, with the objective of identifying the model with the lowest validation loss for the given task of classifying knee OA cases and controls. The same training (*n* = 436) and validation set (*n* = 110) were used for model benchmarking as was used to train DL-binary.

**Fig. 6:**
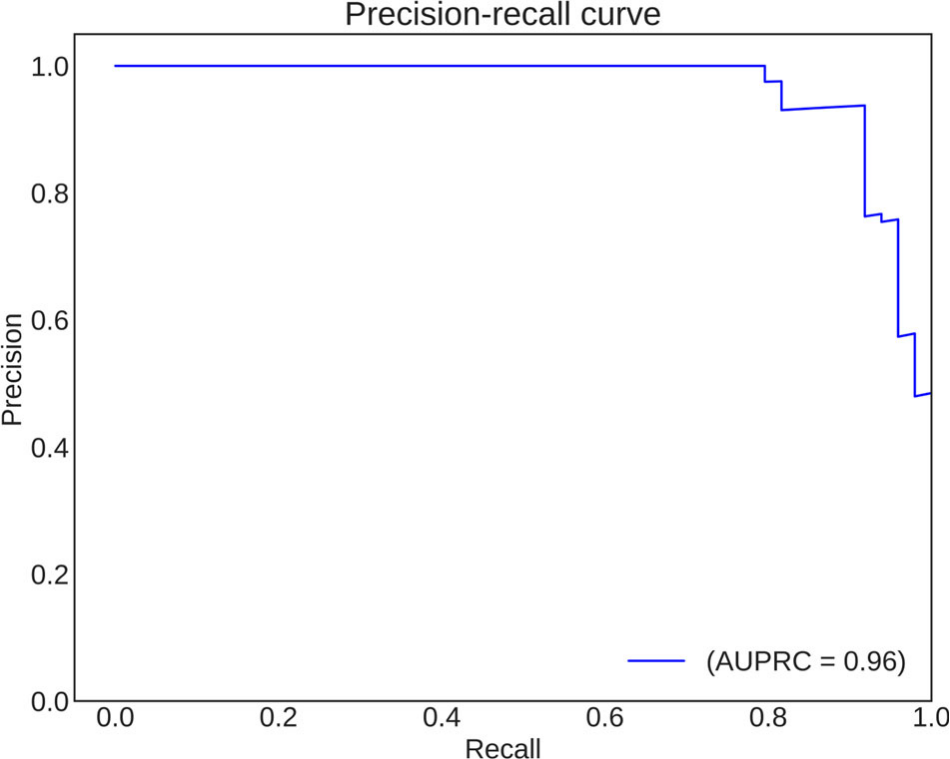
Precision–recall curve for DL-binary predictions on the validation set. Precision–recall curve for the ResNet-101 binary classifier. The curve illustrates the trade-off between precision and recall at varying classification thresholds. The classifier achieved an area under the precision-recall curve (AUPRC) of 0.96, highlighting its ability to effectively separate OA cases from controls.

We compared the performance of six deep-learning architectures, namely VGG-11, VGG-16, VGG-19, ResNet-34, ResNet-50, and ResNet-101, to determine the most suitable model for classifying knee OA cases and controls. All models were trained on the same dataset of knee DXA scans using transfer learning and the same image normalization methods. To ensure a fair comparison, the learning rate (0.003), optimizer (Adam), and the number of epochs trained (70) were kept consistent across all models (Fig. 5). The performance of each model was assessed in terms of accuracy, area under the receiver-operating characteristic curve (AUROC), and area under the precision-recall curve (AUPRC) (Table 2). Among the six models, the ResNet-101 (DL-binary) architecture demonstrated the best performance, with an accuracy of 0.91 and AUROC of 0.96. To understand the effect of class imbalance on our model’s predictions (55% cases and 45% controls in the validations set), we also evaluated the precision-recall curve and AUPRC. The AUPRC was 0.96, highlighting the model’s ability to effectively separate OA cases from controls (Fig. 6).

**Table 2:** Performance metrics of the six deep-learning algorithms in model benchmarking.

Metrics	ResNet-101	ResNet-50	ResNet-34	VGG-19	VGG-16	VGG-11
Accuracy	0.91	0.82	0.87	0.55	0.55	0.61
AUROC	0.96	0.90	0.95	0.59	0.62	0.64
AUPRC	0.96	0.89	0.93	0.49	0.54	0.64

The table presents the validation accuracy, area under the receiver-operating characteristic curve (AUROC), and area under the precision-recall curve (AUPRC) for each algorithm (ResNet-101, ResNet-50, ResNet-34, VGG-19, VGG-16, and VGG-11) used, providing a comprehensive evaluation of their performance in terms of classification accuracy, discrimination ability, and precision-recall trade-off, respectively.

### Binary classification: network architecture and model training

We constructed a ResNet-101 CNN^22^ for our binary DXA image classifier, implementing transfer learning to reduce the amount of training time and resources for our classification task, using a pre-trained model obtained from training on the ImageNet^40^ (image-net.org) dataset and transferring the weights from this model to earlier layers of the network. We applied batch normalization and ReLU after each layer of the CNN to reduce overfitting and provide additional regularization using the Fastai version 1^35^ and PyTorch^34^ default parameters, and dropout was applied to the fully connected portion of the network. The output of the model is a binary classification for each DXA scan-derived image passed in, a one-dimensional tensor containing values of 0 or 1 (control and case status), produced from passing the final layer of the network (the classification head) through the sigmoid and argmax activation functions. The batch size for all models was 64. We first plotted cross-entropy loss as a function of learning rate in order to select the optimal hyperparameters and used the Adam optimizer^41^. We trained the model for 42 epochs with discriminative learning rates ranging from 1 × 10^−3^ to 1 × 10^−6^.

### Binary classification: Grad-CAM analysis for interpretation of model predictions

**Fig. 7:**
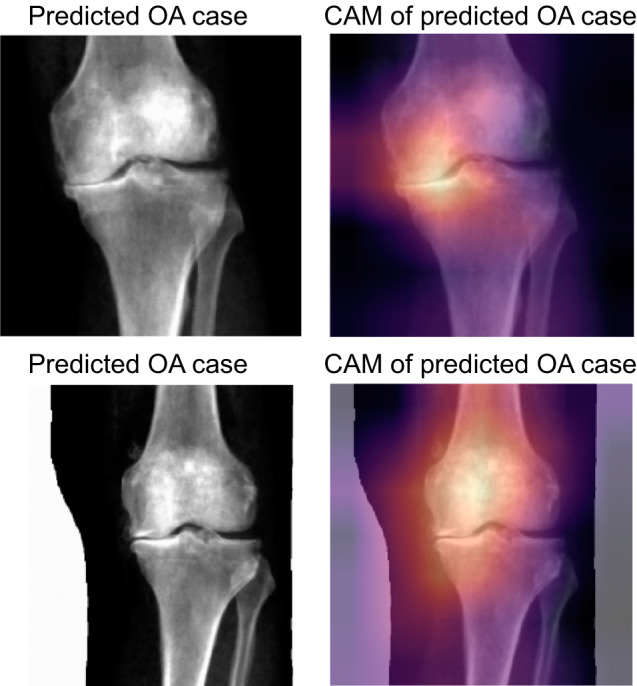
Grad-CAM interpretation of DL-binary predictions. Grad-CAM heat mapping showing areas of highest activation for a control and case prediction made on the knee DXA images. The regions of the map with the darkest color (purple) represent areas that were less informative in the prediction, while lighter colors (yellow) indicate regions of the image that informed the ‘case’ or ‘control’ prediction the most. We find areas of highest activation are most often on the medial side of the knee joint.

To provide a visual interpretation of DL-binary’s predictions on our DXA images, we employed the Gradient-weighted Class Activation Mapping (Grad-CAM) technique. Grad-CAM is a visualization technique that highlights the regions in the input image that have contributed the most to the classifier’s final decision, allowing for better understanding and interpretability of the model’s predictions. The Grad-CAM method generates a heatmap, overlayed on the original input image, indicating the areas that the model considers important for predicting the presence or absence of OA in the knee joint. The heatmap is generated by computing the gradients of the target class score with respect to the feature maps of the last convolutional layer in the ResNet-101 architecture. The gradients are then pooled using global average pooling to obtain the weights for each feature map. These weights are subsequently combined with the feature maps to produce a coarse localization map, which is then upscaled to match the input image size, resulting in the final heatmap. In this work, we used Grad-CAM to visualize the regions in the knee DXAs that were critical for the classifier’s decision-making process (Fig. 7). This allowed us to gain insight into the model’s decision-making process, and confirm that the model was focusing on relevant anatomical features, such as medial compartment knee joint space narrowing or presence of osteophytes.

### Image segmentation: DXA scan annotation procedure

We collected human-generated annotations of each anatomical structure present in 63 DXA scans of the knee (40 training, 23 validation). Annotations were produced at the pixel level for each of the following segments of an AP knee DXA scan the: (1) femur, (2) tibia, and (3) fibula. All annotations were reviewed by an orthopedic surgeon prior to training.

### Image segmentation: network architecture and model training

We trained a U-net architecture^23^ with a 34-layer ResNet encoder^22^ to perform semantic segmentation of the knee joint, annotating the femur, tibia, and fibula coded as 1, 2, and 3, respectively, at pixel-level resolution. We used a batch size of 4 for the segmentation model. We used the same transfer learning approach with the ImageNet dataset as described for the binary classifier, as well as a progressive upsampling strategy during training. First, we downsampled masks to half their size, trained for 28 epochs, saved the model, then restarted the kernel and trained the saved model on regular now upsampled mask. This training procedure was used to efficiently utilize memory and reduce the model’s time to convergence. As described previously, we plotted cross-entropy loss as a function of learning rate in order to select the optimal hyperparameters and used the Adam optimizer^41^.

### Image segmentation: measurement and quality control

After performing segmentation, we computed the minimum inter-bone knee joint space distance in pixels (of either leg), abbreviated as mJSW. Segmentation masks were processed using software developed for this analysis, written in python using the numpy^37^ and opencv-python^38^ libraries. Labeled polygons within each segmentation mask were processed independently, converted to an identity matrix of ones and zeros (ones being the polygon processed, for example, the femur, tibia or fibula). From this identity matrix, two matrices were produced from indexes produced and along the *x* and *y* axes. These indexes were used in the computation of basic features of the polygon such as maximum width, and maximum height. Indices were saved from this process and were later used to compute measurements of joint space width between the femur and tibia.

A major issue in combining our analysis across input pixel ratios was that these pixel ratios represented different resolution scalings due to variable distance between the scanner and the patient as a function of DXA scanner type and the size of the patient. To control for this scaling issue and to standardize the images, we chose to regress our mJSW phenotype measurements across all image resolutions with height obtained from the UKB. The estimates obtained from this regression were used to obtain a scaling factor for each image resolution that were then used for measurement normalization. We validated this regression and normalization procedure by comparing measurements taken on individuals who had DXA scans taken at two imaging assessments at different resolutions.

### Genetic QC

For all genome-wide association analyses, we filtered the participants to Caucasian individuals (FID 22006) from the white, British population (FID 21000) as determined by genetic PCA and participant surveys. We removed individuals whose reported sex (FID 31) did not match genetic sex (FID 22001), had evidence of aneuploidy on the sex chromosomes (FID 222019), were outliers of heterozygosity or genotype missingness rates as determined by UKB quality control of sample processing and preparation of DNA for genotyping (FID 22027), or had more than nine third-degree relatives or any of unknown kinship (FID 220021). In total, 402,233 individuals remained. We further filtered to imaged participants (FID 20158) with complete DXA measurements (FID 12254); 33,475 remained.

Imputed genetic data for 487,253 individuals was downloaded from UKB for chromosomes 1 through 22 (FID 22828) then filtered to the quality-controlled subset using PLINK2^42^. All duplicate single nucleotide polymorphisms (SNPs) were excluded (--rm-dup “exclude-all”) and restricted to only biallelic sites (--snps-only “just-acgt”) with a maximum of 2 alleles (--max-alleles 2), a minor allele frequency of 0.1% (--maf 0.001), individual missingness rates no more than 2.5% (FID 22005), and genotype missingness of no more than 5% (--maxMissingPerSnp 0.05). In total, 14,846,570 SNPs remained in the imputed dataset. Non-imputed genetic data did not contain duplicate or multiallelic SNPs but were filtered to the quality-controlled subset; 703,993 SNPs remained.

### GWAS

GWAS was carried out using PLINK2, with a minor allele frequency of 0.001, a missingness per SNP of 5%, and a missingness per individual of 2.5%. Covariates were the first 20 genetic principal components provided by UKB (FID 22009), sex (FID 31), age (FID 21022), BMI (FID 21001) and standing height taken at the imaging assessment, instance 2 (FID 50). The final population size for all GWAS after both genetic and imaging QC was 29,257, and all GWASs had the same number of SNPs: 12,129,706. SNPs in each resulting GWAS were clumped using --clump with a significance threshold of 5.0 × 10^−8^, a secondary significance threshold of 1.0 × 10^−4^ for clumped SNPs, an *r*^2^ threshold of 0.1, and a 250 kb threshold of physical distance. SNPs were assigned to genes with --clump-verbose --clump-range glist-hg19.

### Annotation of genome-wide significant loci

We carried out a systematic analysis to connect each loci we identified as genome-wide significant with existing literature. First, we identified overlaps of our loci with other traits using the GWAS catalog. We queried the GWAS catalog for any reported associations within a 250 kb region upstream or downstream of each of our loci. The traits that were associated with the most number of these loci were anthropometric traits, namely, height (100% of our independent loci overlapped a height-associated loci in the GWAS catalog). This is to be expected as height has 12,000 genome-wide significant variants that cover about 30% of the human genome with LD-based tagging. In addition to height, heel bone mineral density (44% overlap), BMI-adjusted hip circumference (44% overlap), and appendicular lean mass (44% overlap) were among the traits associated with each of our loci (Table 3). Second, for each locus, we annotated each clumped region with protein-coding genes within 250 kb and queried the Human-Mouse Disease Connection (HMDC) database (Table 4). Third, we also carried out a similar analysis examining rare disease associations for each gene using the Online Mendelian Inheritance in Man (OMIM) database. Four (rs10500759, rs4978572, rs6504045, and rs975889) out of seven loci (or 57%) that mapped only to a single gene resulted in abnormal skeletal phenotypes when disrupted in mice, annotated in the HMDC database. One of these genes, *TBX4*, is a known developmental gene associated with hindlimb development and is also associated with a rare autosomal dominant disease in humans that specifically implicates the patella (annotated in OMIM). Individuals with this disorder have patellae that are small and laterally displaced or dislocated.

**Table 3:** Traits associated with each genome-wide significant loci discovered via GWAS with the mJSW phenotype, as queried by the GWAS catalog.

Phenotype	Associated SNPs	Proportion of total significant SNPs (%)
Height	rs76207439, rs73566656, rs2236996, rs1351266, rs1346, rs34851490, rs7801187, rs10500759, rs55928198, rs11592205, rs6940664, rs4978572, rs9493174, rs6504045, rs975889, rs11049562, rs34195470, rs115710080	100
Lung function (FVC)	rs73566656, rs2236996, rs34851490, rs7801187, rs10500759, rs975889, rs11049562, rs34195470, rs1351266	50
Heel bone mineral density	rs76207439, rs1346, rs7801187, rs10500759, rs975889, rs11049562, rs115710080, rs1351266	44
Appendicular lean mass	rs2236996, rs1346, rs7801187, rs10500759, rs6504045, rs11049562, rs34195470, rs1351266	44
Hip circumference adjusted for BMI	rs2236996, rs1351266, rs1346, rs7801187, rs10500759, rs6940664, rs11049562, rs115710080	44
Protein quantitative trait loci (liver)	rs76207439, rs73566656, rs34851490, rs7801187, rs10500759, rs6504045, rs975889, rs34195470	44

The second column lists the SNPs that were associated with that particular trait, and the third column lists the proportion of the total SNPs discovered that were associated.

**Table 4:** Phenotypes in mouse models as sourced from the HMDC database associated with novel loci that mapped to a single gene.

Gene	Mouse phenotype	Human disease
*CREB5*	Mortality, aging	None
*TEAD1*	Adipose tissue cardiovascular system craniofacial embryo growth, size, body homeostasis, metabolism mortality, aging muscle nervous system skeleton vision, eye	Sveinsson chorioretinal atrophy
*UST*	Behavior, neurological mortality, aging	None
*CCDC91*	None	None
*COL27A1*	Cardiovascular system craniofacial embryo growth, size, body hematopoietic system homeostasis, metabolism immune system limbs, digits, tail mortality, aging renal, urinary system reproductive system respiratory system skeleton	None
*TBX4*	Cardiovascular system, cellular embryo growth, size, body homeostasis, metabolism limbs, digits, tail mortality, aging skeleton	Arthropathy ischiocoxopodopatellar syndrome
*MKX*	Limbs, digits, tail muscle skeleton	None

In addition to associated mouse phenotypes, the third column of the table lists human diseases associated with each gene as obtained from OMIM.

### Heritability and genetic correlation

LD Score v1.0.1 was used to compute linkage disequilibrium regression scores per chromosome with a window size of 1 cM^24^ with the non-imputed genetic data. The heritability of each phenotype was then assessed using LD score regression^24^ with the same covariates as the GWAS. We examined the pairwise genetic correlation of the DL-binary and mJSW model phenotypes using GCTA version 1.93.2 beta for Linux^43^. We created the genetic relationship matrix for our quality-controlled subset with a minor allele frequency of 0.001, and then ran GCTA, using the first 20 genetic principal components provided by UKB (FID 22009), sex, age, BMI, and standing height as covariates.

### Polygenic risk scoring and logistic regression

PRSs were computed with the IDP GWAS summary statistics in PLINK (v1.9) using the clumping and thresholding method. GWAS were clumped using an *r*^2^ threshold of 0.1 and a 250 kb threshold of physical distance for clumping. Significance thresholds of 1, 0.1, 1 × 10^−2^, 1 × 10^−3^, 1 × 10^−4^, 1 × 10^−5^, and 1 × 10^−6^ were used to compute PRSs for all three phenotypes run in GWAS. We then regressed ICD-10 code diagnosis of knee OA on the z-scores generated from each PRS obtained for each phenotype in all genotyped non-imaged individuals of white British ancestry (who had also undergone genetic QC), n = 371,723. In our logistic regressions, we controlled for age, sex, height, BMI, and the first 20 principal components as covariates for all phenotypes. We also control for steroid medication usage that are commonly prescribed to treat inflammation, reduce pain, swelling, and stiffness in joints and other tissues (Table 5), and coded participants that had been prescribed any of the above generic corticosteroids, as a “1”, and “0” otherwise. In order to adjust for past knee trauma, we examined whether individuals had evidence for patella fracture (FID 20002, data coding: 1650) or soft tissue inflammation including Tendonitis (FID 20002, data coding: 1619), Synovitis (FID 20002, data coding: 1621) and bursitis of the knee (FID 20002, data coding: 1624). In our model, we coded participants that had any of the following in the EHR as a “1”, and “0” otherwise.

**Table 5:** Prescription steroid medications and their corresponding data coding numbers (UK Biobank FID 20003).

Medication	Data coding
Prednisone	1140868364
Prednisolone	1140874930
Methylprednisolone	1140874976
Triamcinolone	1140868426
Hydrocortisone	1140874896
Dexamethasone	1140874816
Betamethasone	1140874790

The table lists commonly used corticosteroids in orthopedics for the treatment of joint inflammation and various inflammatory conditions. The medications include oral, injectable, and topical formulations, demonstrating the diverse range of administration methods to address patient-specific needs and preferences.

## Data Availability

All data used for this study were obtained from the UK Biobank under application number 65439. GWAS summary statistics are available at https://utexas.box.com/s/pc5wbzvvaf42pegzww0u7k18wovtri97.

## Code availability

Deep-learning and image processing tools can be found at https://github.com/briannaflynn/UKB_knee_segmentation and https://github.com/briannaflynn/dxaconv/. Individual-level information of image-derived phenotypes has been reported back to the UKB and will be available upon publication.
